# Observations in Support of Equivocal Generation

**Published:** 1811-12

**Authors:** 


*To the Editors of the Medical and Physical Journal.*

GENTLEMEN,

OBSERVING a paper in your last, concerning the generation of Worms, I shall beg leave to state the following experiments, as having, I think, some relation to that long exploded doctrine, equivocal generation. After having read Mr. Hamilton's theory, to satisfy myself I took a small piece of buttock of beef, out of the inside, and put it into a bottle with a dram or two of distilled water upon it, merely to keep it from drying. I then stopped it completely down, and placed it in the heat of about 70. After suffering it to remain nearly a week, I opened it; there was a great deal of air extricated, and the meat was abominably fœtid. I examined a portion of it by means of a microscope, and found that there were a number of small worms or maggots in it, which were not perceptible to the naked eye. When I had finished this experiment, I was not perfectly satisfied, and therefore determined upon another. I had often observed a sort of small worm floating in vinegar, commonly known by the name of vinegar eels. I endeavoured to purify some of this vinegar by first boiling it, and then passing it through a filter: after having done this, I examined it by the microscope, and found it peefectly free from any thing of the kind. I filled a vial with some of this, stopped it, and set it by for a few days; when, upon examination, I found the same appearance as there was before I purified it, only that the worms were smaller. I was now perfectly satisfied that there was such a thing as equivocal generation; and it is upon this foundation that I suppose worms may be produced in any part of the body, by diseased matter being thrown out and entering into the putrefactive fermentation. If Mr. Hamilton, or any other of your readers, can give me reason to believe that these worms were not produced without ova, I shall be open to conviction; and they will greatly oblige your humble Servant,

MEDICUS.

*October* 14, 1811.

